# The Emerging Role of MicroRNAs in Breast Cancer

**DOI:** 10.1155/2020/9160905

**Published:** 2020-07-03

**Authors:** Zhiguang Yang, Zhaoyu Liu

**Affiliations:** Department of Radiology, Shengjing Hospital of China Medical University, Shenyang, Liaoning 110004, China

## Abstract

Breast cancer (BC) is the most common malignancy in women. Due to BC heterogeneity, complexity, and metastasis, many BC patients do not successfully respond to therapies. The effective management of BC depends on early diagnosis and monitoring of drug response. Therefore, identifying new biomarkers for the diagnosis, prognosis, and development of new drugs is urgently required. Dysregulation of microRNAs (miRNAs) participates in the tumorigenesis and progression of cancers, especially breast cancer (BC). Several studies demonstrated that miRNAs could perform their function as oncogenes or tumor suppressors. This review describes recent progress on the role of microRNAs in the diagnosis, prognosis, hallmark, and treatment of BC. According to a recent literature survey, miRNAs play a pivotal role in the regulation of hallmarks of cancer, such as proliferation, apoptosis, invasion, metastasis, and tumor stemness. Many miRNAs are potential biomarkers for BC for diagnosis, and some are indicators of prognosis. Moreover, circulating miRNA profiles, as minimally invasive, diagnostic, and prognostic markers, are broadly used in BC therapy, and some miRNAs are good predictors of therapeutic outcomes. Other miRNAs are involved in overcoming chemoresistance and in increasing BC drug sensitivity.

## 1. Introduction

Breast cancer (BC) is one of the most common malignant tumors and the second leading cause of cancer in women [1]. Approximately, 1.5 million new cases are annually diagnosed with breast cancer [2] and almost 460,000 patients died each year due to BC chemoresistance and metastasis. BC biological characteristics are routinely used for early detection, prognosis, and selection of the therapeutic strategy, including histologic subtype, grade [3], lymph node status, hormone receptor, and human epidermal growth factor receptor 2 (HER2) statuses [4]. Some of the mentioned characteristics are related to patients' survival and posttreatment clinical outcomes [5]. However, several BC patients, who had similar characteristics, showed different clinical outcomes. Therefore, biological features have limitations with regard to diagnosis, prognosis, and clinical outcomes' prediction [6]. Thus, novel diagnostic and prognostic approaches are urgently required for the identification of new personalized therapeutic methods that improve BC patients' quality of life.

MicroRNAs (miRNAs) are a group of small noncoding RNAs that can interrupt the expression of protein-coding genes by binding to their mRNAs and inhibiting therefore their protein translation [7]. So far, an estimated total of 28,000 mature miRNAs was reported to participate in posttranscriptional regulation during cellular processes, including cancer cell proliferation, differentiation, migration, apoptosis, and angiogenesis. miRNA abnormal expressions are also considered to be potential biomarkers of BC as they are stably detected in tumor tissues [8] and in patients' body fluids, including blood, serum, plasma, and saliva [9, 10]. miRNAs in body fluids, also called circulating miRNAs, are remarkably stable, packaged into extracellular microparticles or bound with lipoproteins, which protect them from RNase digestions [10, 11]. miRNA profiles have been effectively used to classify BC patients as treatment responding or nonresponding groups [12]. Therefore, miRNAs have been clearly demonstrated to potentially regulate BC progression and used as new diagnostic, prognostic, and predictive biomarkers of BC [6].

In this review, we summarize recent publications on miRNA functions in the regulation of BC progression and discuss the clinical potential of miRNAs as biomarkers of early and differential diagnosis and prognosis, and as determinants of chemoresistance and therapy selection. Some BC therapeutic strategies that involved miRNAs are reviewed, which provides new insights into breast cancer therapy.

## 2. miRNAs and Breast Cancer

miRNAs are a class of 18 to 24 nucleotides noncoding regulatory RNAs [13], which specifically target mRNA 3′-untranslated regions (3′UTRs) leading to their translational repression and/or mRNA decay, degradation, or deadenylation [14]. An important characteristic of miRNAs is their capacity to bind to more than a hundred mRNAs' 3′UTRs [15]. Interestingly, one transcript can be regulated by various miRNAs [16]. In the human genome, an estimated 60% of genes can be recognized by different miRNAs [17]. miRNAs participate in the regulation of essential biological processes and several diseases. There is a long history about regulatory relationships between miRNAs and hallmark of cancer, especially breast cancer. As early as 2005, Lu et al. reported the differential expression of miRNAs in breast cancer [18]. Subsequently, an increasing number of studies have suggested that miRNAs are closely associated with BC occurrence and development. miRNAs were reported to play two important roles as oncogenes (onco-miRNAs) and tumor suppressors.

### 2.1. Onco-miRNAs in Breast Cancer

Many miRNAs targeting tumor-suppressor genes are overexpressed in BC. These miRNAs regulate the tumorigenesis, proliferation, invasion, and migration of cancer cells [19]. miR-10b is highly expressed in early metastatic and recurrent BC patients [20] and is associated with increased proliferation, migration, and invasion of BC cells via E-cadherin targeting [21]. miR-21 was demonstrated to promote the transformation and development of BC via suppressing the programmed cell death protein 4 (PDCD4) expression [22]. miR-155 acts as an onco-miRNA in BC, which inhibition by an antisense oligonucleotide remarkably prevented proliferation and induced cell apoptosis [23, 24]. Moreover, miR-200a has been shown to suppress apoptosis of BC cells by targeting the transcriptional regulator yes-associated protein 1 (YAP1) [25]. Finally, miR-27b targeted the ST14 (suppression of tumorigenicity 14) gene and enhanced the invasion and migration of breast cancer cells. All the discussed miRNAs are summarized in Table 1.

### 2.2. Tumor-Suppressor miRNAs in BC

Tumor-suppressor miRNAs are usually downregulated in cancer cells. They can inhibit cancer progression via silencing oncogenes and tumor-promoting genes. Many miRNAs, such as lethal-7 family (let-7), miR-26b, miR-124, miR-125a/125b, miR-205, and miR-206, were reported to act as tumor-suppressor miRNAs. miR-26b prevents the tumorigenesis of triple-negative breast cancer (TNBC) cells by targeting DEP domain containing 1(DEPDC1) and downregulating FOXM1 expression [27]. miR-26b was identified to facilitate G0/G1 cell cycle arrest and to inhibit cellular proliferation via CDK8 targeting [28]. Ma et al. found that miR-26a/26b could inhibit BC progression through inhibiting the expression of ST8 alpha-N-acetyl-neuraminide alpha-2,8-sialyltransferase 4 (ST8SIA4) [29]. miR-124-3p was reported to be downregulated in BC cells, where it regulates their proliferation and invasion by targeting MGAT5 [30]. miR-205 downregulation enhanced BC bone metastasis and invasion by targeting TG (transglutaminase) 2 [31]. miRNA-205 could also partially decrease the survival of TNBC cells and epithelial-mesenchymal transition (EMT) by targeting the HMGB1-RAGE signaling pathway [32]. Sheng-Nan et al. reported that miR-21-3p expression could be stimulated by berberine, which inhibited MCF-7 proliferation via targeting cytochrome P450 1A1 (CYP1A1) [33]. miR-628 inhibited the migration and invasion of BC cells by targeting SOS1 [34], which suggested that therapeutic strategies that increase its expression may be an effective treatment approach against BC metastasis [35]. All the discussed miRNAs are summarized in Table 2.

## 3. miRNAs as BC Diagnostic Biomarkers

Breast Cancer is a type of heterogeneous diseases presenting multiple morphological appearances, molecular features, phenotypes, and therapeutic responses [37, 38]. BC therapeutic strategies are dependent on the availability of reliable diagnostic, prognostic, and predictive factors that direct the determination and selection of the appropriate treatments [39]. Circulating miRNAs were reported to be good biomarkers of BC diagnosis. For instance, the circulating miRNAs, such as let-7a, miR-10b, and miR-155, were identified to be highly expressed in melanoma, breast, prostate, colon, and renal cancers. The expression of circulating miR-195 expression is specifically elevated in breast cancer [40]. Circulating miR-21 could be used as a BC biomarker [41]. miR-373 presence in the serum of BC patients was also reported to be a good biomarker [42]. The expression level of circulating miR-16, miR-21, miR-23*α*, miR-146*α*, miR-155, and miR-181*α* may reflect different outcomes in BC [43]. miR-195-5p and miR-495 represent potential circulating molecular markers for BC early diagnosis in minimally invasive surrogate sample sources [44].

miRNAs are used as prognostic biomarker in Breast cancer and accumulating evidence indicated that circulating levels of miRNAs may be associated with the outcomes. Pre-miR-488 expression may be a new prognostic marker that predicts disease recurrence in BC patients [45]. The circulating miR-21 and miR-125B are regarded as new noninvasive prognostic markers for neoadjuvant chemotherapy response and prognosis of BC patients [46].

## 4. miRNAs and Breast Cancer Stem Cells (BCSCs)

Cancer stem cells (CSCs) are defined as “a small subset of the cancerous populations, responsible for tumor initiation and growth, and that possesses the characteristic properties of quiescence, indefinite self-renewal, intrinsic resistance to chemotherapy and radiotherapy, and the capability to give rise to differentiated progenies” [47]. They have also been shown to be responsible of cancer recurrence following chemotherapy. miRNAs were reported to regulate the function of BCSCs by promoting or inhibiting cancer progression. Multiple miRNAs are associated with phenotype of BCSCs. miR-708 was reported to suppress BCSCs' self-renewal; In addition, the miR-708/CD47 axis was proved to be a good target for TNBC treatment [48]. miR-1 can regulate BCSCs' proliferation, apoptosis, and EMT by inhibiting ecotropic virus integration site-1 (Evi-1) [49]. The upregulation of miR-210, induced by hypoxic exposure, promotes BCSCs' migration, proliferation, and self-renewal by targeting E-cadherin [50]. miR-422a attenuated BCSCs' proliferation and tumorigenesis by suppressing the expression of patatin-like protein 2 (PLP2) [51]. miR-221/222 promotes BCSCs' proliferation, migration, and invasion via regulating phosphatase and tensin homolog (PTEN) [52]. All the discussed miRNA are summarized in Table 3.

## 5. miRNAs in Clinical Treatment of Breast Cancer

The use of miRNAs as anticancer treatment strategies has been developed by two methods. The first consists in using miRNAs as pharmaceutical molecules that can increase or decrease miRNA levels in BC based on the synthesis and transmission of specific oligonucleotides. The other is based on regulating miRNAs to improve the efficacy of conventional treatments within combined therapeutic strategies.

Oligonucleotide analogs and antagonists represent two major miRNA therapies. Single-stranded oligonucleotides with miRNA-complementary sequences are used to silence the miRNA function of target proteins. Functional miRNA liposomes have been developed to inhibit SLUG expression and the TGF-*β*1/SMAD pathway in TNBC cells, and to enhance the efficacy of chemotherapy in mice [53]. Epigenetic strategies using histone deacetylase inhibitors or functionally cooperative miRNAs are effective approaches for eliminating HER3 signal transduction for BC treatment [54]. In addition, 2,3,7,8-tetrachlorodibenzo-p-dioxin (TCDD) treatment induces the overexpression of circulating miRNA via miR-3942-3p/BARD1 and prevents the occurrence of BC through blocking the cell cycle and promoting apoptosis [55].

miRNA antagonists can inhibit the function of miRNAs in human disease, and miRNA analogs are used to repair miRNAs with loss of function, similar to traditional gene therapy. This approach is also known as miRNA replacement therapy. This new therapy attracts more interests as it may provide a new opportunity to develop tumor inhibitors. Moreover, anti-miRNA oligonucleotides (AMOs) [56], locked nucleic acid (LNA)-modified oligonucleotides [57], cholesterol-binding anti-miRNA molecules [58], and 2′-O-methoxyethyl-4′-sulfur RNA (MOE-SRNA) [59] were successfully used to improve transmission efficiency of miRNA or anti-miRNA molecules *in vivo*. In addition, modified high-efficiency miRNA molecules with long half-lives have also been developed. miRNA mimics or antagomirs were used in nanomaterials' packages, especially gold nanoparticles, to promote drug efficiency [60–62]. The DTX (docetaxel)/miR-34a nano cocarrier is a new nanoplatform that integrates insoluble drugs and gene/protein drugs, which provides a promising strategy for the treatment of metastatic BC [63].

### 5.1. miRNAs and BC Chemotargeting

Some methods have been studied for miRNA application as a medical targeting in cancer therapy, including the use of miRNA mimics, which are imitations of increased miRNAs, or antagomirs, which inhibit onco-miRNAs in cancer cells [64]. Gilam et al. identified that miR-96/miR-182 delivery could prevent metastatic breast cancer via targeting palladin in a preclinical model [65]. miR-145 expression is downregulated in BC cells, which is proved to be therapeutic targeting of DLD-1 and SW480 [66]. ANKRD46 was recently identified as a direct target of onco-miRNA miR-21 in BC, and thus, its inhibition using peptide nucleic acid (PNA) anti-miR-21 provides potential therapeutic applications in BC treatment [67].

### 5.2. miRNAs and BC Chemoresistance

miRNAs disturbance leads to BC chemoresistance, such as the case for miR-125b. miRNA oligonucleotide analogs have the potential to increase the level of specific miRNAs that are lost in BC and to elevate the sensitivity of drugs. The combination with conventional and miRNA therapies could improve the prognostic benefits for BC patients. Yang et al. reported that an miRNA oligonucleotide analog of miR-195 increased drug sensitivity in adriamycin-resistant BC cells, by downregulating RAF-1 and BCL-2 expression resulting in apoptosis induction [68]. Anti-miRNA oligonucleotides may also be used to suppress specific miRNA expression in BC patients by enhancing the efficacy of conventional treatment. For example, anti-miR-21 oligonucleotides can kill BC cells by their binding to HER2, which promote trastuzumab sensitivity [69]. Furthermore, miR-145 mimics promote BC doxorubicin sensitivity by targeting the multidrug resistance-associated protein-1 [70]. miR-125b-5p overexpression increases tamoxifen sensitivity in BC cells [71]. Furthermore, miR-221/222 expression has been related to tumor tolerance to tamoxifen therapy [72]. miR-451 mimic transfection improves the sensitivity of MCF-7/DOX (doxorubicin)-resistant cells to DOX, which indicates that restoring miRNA expression has the potential of overcoming drug resistance in cancer cells [73]. Pan et al. demonstrated that miR-328 regulates ABCG2 expression, which altered drug resistance of BC cells [74]. miR-346, miR-181a, miR-638, miR-211, miR-212, miR-216, miR-199b, miR-204, miR-328, miR-373, miR-424, and miR-768-3p regulate fulvestrant resistance in BC via the TGF-*β* signaling pathway [75]. Zhang et al. [76] demonstrated that miR-100 is associated with paclitaxel sensitivity in BC. In addition, miR-140-5p can enhance the doxorubicin sensitivity of BCSCs via the WNT1/ABCB1 pathway [77]. Therefore, miRNAs play an important role in regulating the chemoresistance of breast cancer cells.

## 6. Discussion

In this review, we reviewed that miRNAs play important roles in regulating BC progression and their clinical value in the diagnosis, prognosis, and therapy of BC patients. An increasing number of studies are reported on the prognostic and diagnosis values of circulating miRNAs or tissue specific miRNAs in breast cancer patients. Based on our review, microRNAs have potential for clinical diagnosis and prognosis. Thus, miRNAs are becoming new diagnostic and prognostic biomarkers for BC and may help predict the response of tumors to specific chemotherapeutic agents. Therefore, these published studies about miRNA as biomarkers could provide a convincing conclusion on their clinical application. There are many factors influencing the data provided by the different studies, including differences in sample preparation, small sample numbers, different miRNA experimental methods (qPCR or different miRNA array platforms), and treatment/tumor heterogeneity. Further studies on more homogeneous populations are needed to identify these miRNAs for BC diagnosis.

The potential of miRNAs should not be limited to their use as biomarkers for BC. We summarized recent progress on the role of miRNAs in controlling specific cellular processes in BC, such as invasion, migration, proliferation, and apoptosis. miRNAs also act as tumor suppressors or oncogenic miRNAs that facilitate BC occurrence, development, and metastasis; therefore, future studies may focus on the development and delivery of miRNA drugs for BC. Although there has been success with some miRNAs, such as miR-9 [78] and miR-21 [69], particular attention should be paid to optimize the stability, delivery, and effectiveness of targeted therapies in miRNA drug control. Because miRNA therapy can regulate the treatment response, the combination of non-miRNA and miRNA therapies is strongly recommended to improve the efficacy of the non-miRNA therapies (e.g., chemotherapy) for BC. In summary, the investigation of miRNA molecular mechanisms in the regulation of breast cancer tumorigenesis or progression may provide novel therapies of BC.

## Figures and Tables

**Table 1:** List of some onco-miRNAs.

miRNA annotation	Function	Target	Ref.
miR-10b	Increases the proliferation and promotes the migration and invasion of BC cells	E-cadherin	[21]
miR-21	Promotes the transformation and development of BC	PDCD4	[22]
miR-155	Increases the proliferation and inhibits cell apoptosis of BC cells	FOXO3a	[23, 24]
miR-200a	Suppresses apoptosis	YAP1	[25]
miR-27b	Enhances invasion and migration	ST14	[26]

This table provides examples of miRNAs described in the text, with a particular attention on their targeting and function.

**Table 2:** List of some tumor-suppressor miRNAs.

miRNA annotation	Function	Target	Ref.
miR-206	Prevents TNBC tumorigenesis	DEPDC1	[27]
miR-26b	Facilitates G0/G1 cell cycle arrest and inhibits cellular proliferation	CDK8	[28]
miR-26a/26b	Inhibits BC progression	ST8SIA4	[29]
miR-124	Inhibits BC cell proliferation and invasion	STAT3/MGAT5	[30, 36]
miR-205	Reduces BC bone metastasis and invasion	TG2	[31]
miR-21-3p	Inhibits BC cell proliferation	CYP1A1	[33]
miR-628	Inhibits the migration and invasion of BC cells	SOS1	[35]

This table provides examples of miRNAs described in the text, with a particular attention on their targeting and function.

**Table 3:** List of some miRNAs involved in the BCSC phenotype.

miRNA annotation	Function	Target	Ref.
miR-708	Suppresses BCSCs' self-renewal	CD47	[48]
miR-1	Regulates BCSCs' proliferation, apoptosis, and EMT	Evi-1	[49]
miR-210	Promotes BCSCs' migration, proliferation, and self-renewal	E-cadherin	[50]
miR-422a	Attenuates BCSCs' proliferation and tumorigenesis	PLP2	[51]
miR-221/222	Promotes BCSCs' proliferation, migration, and invasion	PTEN	[52]

This table provides examples of miRNAs described in the text, with a particular attention on their targeting and function.

## Additional Points

*Highlights*. MicroRNAs can regulate the hallmarks of breast cancer (BC). MicroRNAs are good biomarkers in BC diagnosis and prognosis. MicroRNAs play an important role in BC drug resistance. MicroRNAs may be a good therapeutic targeting approach for BC.
